# KSRP Deficiency Attenuates the Course of Pulmonary Aspergillosis and Is Associated with the Elevated Pathogen-Killing Activity of Innate Myeloid Immune Cells

**DOI:** 10.3390/cells13242040

**Published:** 2024-12-10

**Authors:** Vanessa Bolduan, Kim-Alicia Palzer, Frederic Ries, Nora Busch, Andrea Pautz, Matthias Bros

**Affiliations:** 1 Department of Dermatology, University Medical Center of the Johannes Gutenberg University, 55131 Mainz, Germany; 2 Department of Pharmacology, University Medical Center of the Johannes Gutenberg University, 55131 Mainz, Germany; 3 Department of Hematology and Medical Oncology, University Medical Center of the Johannes Gutenberg University, 55131 Mainz, Germany

**Keywords:** innate immunity, RNA-binding proteins, invasive pulmonary aspergillosis, phagocytosis, neutrophils, macrophages

## Abstract

The mRNA-binding protein KSRP (KH-type splicing regulatory protein) is known to modulate immune cell functions post-transcriptionally, e.g., by reducing the mRNA stability of cytokines. It is known that KSRP binds the AU-rich motifs (ARE) that are often located in the 3′-untranslated part of mRNA species, encoding dynamically regulated proteins as, for example, cytokines. Innate myeloid immune cells, such as polymorphonuclear neutrophils (PMNs) and macrophages (MACs), eliminate pathogens by multiple mechanisms, including phagocytosis and the secretion of chemo- and cytokines. Here, we investigated the role of KSRP in the phenotype and functions of both innate immune cell types in the mouse model of invasive pulmonary aspergillosis (IPA). Here, KSRP^−/−^ mice showed lower levels of *Aspergillus fumigatus* conidia (AFC) and an increase in the frequencies of PMNs and MACs in the lungs. Our results showed that PMNs and MACs from KSRP^−/−^ mice exhibited an enhanced phagocytic uptake of AFC, accompanied by increased ROS production in PMNs upon stimulation. A comparison of RNA sequencing data revealed that 64 genes related to inflammatory and immune responses were shared between PMNs and MACs. The majority of genes upregulated in PMNs were involved in metabolic processes, cell cycles, and DNA repair. Similarly, KSRP-deficient PMNs displayed reduced levels of apoptosis. In conclusion, our results indicate that KSRP serves as a critical negative regulator of PMN and MAC anti-pathogen activity.

## 1. Introduction

KSRP (K homology [KH]-type splicing protein) is an mRNA-binding protein (RBP) that has been demonstrated to limit mRNA stability through direct binding to AU-rich elements (ARE) located within the 3′-untranslated region (3′-UTR) of target mRNAs [1,2,3,4]. Moreover, it inhibits the translation of target mRNAs [5], serves as a transcription [6] and splicing factor [7], and functions as a maturation factor for various microRNA species [8,9,10,11,12,13]. To date, KSRP has been identified as a crucial negative regulator of inflammatory immune responses to avoid excessive immune reactions, such as cytokine storms [14], extensive tissue damage [15], or autoimmune responses [16]. However, the precise role of KSRP within the immune system remains to be determined [17].

The innate immune response acts as a rapid, nonspecific first line of defense, eliminating pathogens, initiating inflammation, recruiting immune cells, and activating the adaptive immune response. When pathogens breach epithelial barriers, they are recognized by phagocytic cells, primarily macrophages (MACs) and polymorphonuclear neutrophils (PMNs) [18,19]. Both MACs and PMNs initiate immune responses by recognizing pathogens via receptors like Toll-like receptors (TLRs), triggering proinflammatory cytokine production [20], recruiting immune cells to sites of infection and inflammation [21], and exhibiting pathogen-killing activities through phagocytosis and the production of ROS [22,23]. Also infiltrate monocytes, differentiating into MACs, appear to be of significant importance in this pathogen-killing process [24].

*Aspergillus fumigatus* (*A. fumigatus*) is a ubiquitous saprophytic fungus that constitutes the predominant airborne fungal pathogen affecting patients with an immunocompromised immune system [25]. In these patients, *A. fumigatus* may cause invasive pulmonary aspergillosis (IPA) after the inhalation of airborne conidia, which germinate in the lungs and sprout there as hyphae [26]. The innate immune system is regarded as being primarily responsible for clearing conidia and preventing the growth of *A. fumigatus* conidia (AFC) [27]. For this, the recruitment of PMNs is essential [28,29]. However, lung-resident leukocytes, including MACs, also initiate an innate counterresponse in the course of IPA [30]. PMNs and MACs eradicate AFC by phagocytosis [31].

In line with the pivotal function of KSRP in inhibiting the expression of proinflammatory mediators, our recent findings indicate that MACs lacking KSRP exhibit a more pronounced inflammatory response in an LPS-induced sepsis model [32]. In this study, we asked whether the stimulation-induced hyperactivation of innate immune cells would also affect the pathogen-eradicating functions of PMNs and MACs in a model of aspergillosis.

We observed reduced AFC levels in KSRP-deficient mice in an in vivo IPA model, along with higher frequencies of PMNs and MACs in the lungs. Somewhat surprisingly, PMN-depleted KSRP-deficient mice survived IPA. Interestingly, a comparison of MAC and PMN RNA sequencing revealed a moderate number of genes equally upregulated in KSRP-deficient PMNs and MACs in response to stimulation, mainly involved in immune responses. Consistent with this, the PMNs and MACs of KSRP^−/−^ mice displayed a higher phagocytic uptake of AFC, and PMNs generated more ROS following stimulation. The majority of PMN-specific upregulated genes were related to the control of the cellular metabolism, DNA repair, and the cell cycle, and we detected reduced levels of apoptotic KSRP-deficient PMNs upon stimulation. In conclusion, our results demonstrate that KSRP limits PMN and MAC anti-pathogen activity.

## 2. Materials and Methods

### 2.1. Mice

KSRP^+/−^ mice (C57BL/6 background) [33] were kept in the Central Animal Facility of Johannes Gutenberg University, Mainz, under pathogen-free conditions. The KSRP^+/−^ mice were mated to obtain KSRP^−/−^ (KO) and WT animals. The mice were genotyped by PCR (primers: KSRP-WT-for GCGGGGAGAATGTGAAGG, KSRP-KO-for CTCCGCCTCCTCAGCTTG, and KSRP-WT/KO-rev GAGGCCCCTGGTT-GAAGG). Mice handling was conducted in accordance with institutional guidelines and was approved by the Rhineland-Palatinate National Investigation Office (approval IDs: G23-1-037). Mice (8–14 weeks of age) of either gender were used for all experiments.

### 2.2. A. fumigatus Strains

The WT and green fluorescent protein (GFP)-expressing (AfS148) *A. fumigatus* strains [34] were cultivated in an *Aspergillus* minimal medium (AMM) [35]. In brief, the conidia were incubated on AMM agar plates (4 d, 37 °C, 5% CO_2_) in order to facilitate fungal growth. The plates were rinsed with water containing glass beads (ø 4 mm; Carl Roth, Karlsruhe, Germany) to facilitate the release of conidia from the plate surface. A sterile 40 μm nylon mesh was used to filter the spore suspension twice. Conidia were kept in water at 4 °C.

### 2.3. Invasive Aspergillosis

The WT and KSRP^−/−^ mice were treated with a mixture of 14.5% ketamine (50 mg/mL) and 5.7% xylazine (0.2%) before receiving 10^7^ AFC administered intratracheally, as previously described [36,37]. Briefly, an indwelling venous catheter (22G; Vasofix, B. Braun AG, Melsungen, Germany) was slid into the trachea, and a fungal suspension (100 µL) was delivered into the trachea. The mice were mechanically ventilated (250 breaths/min, 300 µL of tidal volume, 2 min) using an animal respirator (MiniVent, Hugo Sachs, March-Hugstetten, Germany) to improve lung dispersion, as previously described [35]. Three mice per group were sacrificed 24 h post-infection to assess the early anti-fungal immune response. In another group (three mice), the course of infection was monitored for 2 weeks on a daily basis by clinical scoring (weight, activity, breathing, posture, skin and fur condition). Mice showing severe symptoms were euthanized in accordance with animal ethical guidelines. Where indicated, PMNs were depleted by the intraperitoneal injection (i.p.) of an α-Ly6G antibody (150 µg, clone 1A8; BioXCell, Lebanon, NH, USA) 1 d before inoculation with the AFC suspension.

### 2.4. Immune Cells

Spleen cell suspensions were obtained (Figure S1) via mechanical homogenization, as previously described [35], and washed twice with pre-cooled phosphate-buffered saline (PBS), and red blood cells (RBC) were lysed with hypotonic buffer (155 mM NH_4_Cl, 10 mM of KHCO_3_, and 10 μM of EDTA at pH 7.4). Subsequently, 2 × 10^6^/mL of spleen cells were seeded in 1 mL of an IMDM-based culture medium supplemented with 5% fetal bovine serum (PAN-Biotech, Aidenbach, Germany), 50 μM of ß-mercaptoethanol, 2 mM of L-glutamine, 100 U/mL of penicillin, and 100 μg/mL of streptomycin (all components from Sigma-Aldrich, Deisenhofen, Germany). The spleen cells were left untreated or were stimulated with 1 µg/mL of LPS and 1 × 10^6^ AFC overnight (16 h). Afterwards, the cell culture supernatants were collected and analyzed for cytokine content (see Section 2.6).

Following IPA induction, the lung tissue, the lung-associated lymph nodes, and the BALF were subjected to a flow cytometric analysis (see Section 2.5). To this end, lung tissue was cut and small pieces were digested using 6 mg/mL of collagenase IV plus 0.5 mg/mL of DNase I (diluted in RPMI media) in a thermo shaker for 1 h at 37 °C. Then, 10 mM of EDTA was applied, and samples were incubated for 2 min at 4 °C. The cell suspension was filtered using a 70 µm cell strainer and washed with PBS (8 min, 300× *g*, 4 °C), and erythrocytes were lysed using hypotonic buffer (see above). The lymph nodes were mashed through a 40 µM cell strainer and washed with PBS, following which, the cells were seeded in 96-well plates. The BALF was collected by rinsing the trachea with 600 µL of cold PBS. Next, the BALF was centrifuged to sediment cells. The fluid was used for a cytokine content analysis (see Section 2.6), and the cells of the BAL were seeded into 96-well plates for a flow cytometric analysis (see Section 2.5).

PMNs were isolated from the bone marrow of the mice using biotin-labeled Ly6G-specific antibodies and streptavidin-conjugated beads (both from Miltenyi Biotec, Bergisch Gladbach, Germany), in accordance with the manufacturer’s recommendations. Freshly isolated PMNs were used for different assays (see Section 2.10, Section 2.11 and Section 2.12).

### 2.5. Flow Cytometric Analysis

For the flow cytometric analysis, freshly isolated or differentially pretreated cells were seeded into 96-well culture plates and washed (PBS/2% FCS), and nonspecific antibody binding via Fc receptor engagement was blocked by applying rat anti-mouse CD16/CD32 antibodies (clone 2.4G2). Then, antibodies (Table 1) were added, and the samples were incubated for 20 min. The incubation steps were performed at 4 °C in the dark. All the antibodies were purchased from Thermo Fisher (Waltham, MA, USA) and Biolegend (San Diego, CA, USA). Viability was monitored using a fixable viability dye (FVD), coupled with APC eFluor780 or eFlour450 (Thermo Fisher, Waltham, MA, USA), as recommended by the manufacturer. The samples were analyzed using an Attune™ NxT AcousticFocusing Flow Cytometer, equipped with Attune™ NxT software (version 3.2.1526.0; Thermo Fisher, Waltham, MA, USA).

### 2.6. Cytometric Bead Array

Cytokine concentrations were assessed by a multiplex bead-based immunoassay (LEGEND-plex Mouse Anti-Virus Response Panel; 13-plex, BioLegend, San Diego, CA, USA), as recommended by the manufacturer. Samples were assayed by flow cytometry, (see above) and data were analyzed using LEGENDplex^TM^ Qognit (v8.0, BioLegend, San Diego, CA, USA).

### 2.7. RNA Isolation

The RNA of the IPA-treated lung tissue was prepared by guanidinium isothiocyanate (GIT) chloroform extraction, as previously described [38]. The total RNA was isolated from the PMNs using the RNeasy Plus Mini Kit (Qiagen, Hilden, Germany). The RNA of the tissue and the PMNs was used for real-time PCR, and PMN-derived RNA was also used for bulk sequencing.

### 2.8. Real-Time qPCR

The cDNA was synthesized from the isolated total RNA using the iScript kit (Bio-Rad, Munich, Germany). PMN-derived cDNA was subjected to real-time PCR using primers to amplify *IL-6* (5′-CCGGAGAGGAGACTTCACAG-3′, 5′-CAGAATTGCCATTGCACAAC-3′) and *TNF-a* (5′-CCACCACGCTCTTCTGTCTA-3′, 5′-AGGGTCTGGGCCATAGAACT-3′). Tissue-derived cDNA was analyzed using *TEF1* (5′-CCATGTGTGTCGAGTCCTTC-3′, 5′-GAACGTACAGCAACAGTCTGG-3′). In all cases, expression levels were normalized to *GAPDH* (5′-CCATCACCATCTTCCAGGAG-3′, 5′-TTTCTCGTGGTTCACACCC-3′), serving as a housekeeping gene. The primers were purchased from Eurofins Scientific (Luxembourg City, Luxembourg). The qPCR samples consisted of 200 ng of cDNA, 70 nM of either primer, and 12.5 µL of 2× primaQUANT Master Mix high ROX (Steinbrenner Laborsysteme, Wiesenbach, Germany). The samples were tested in technical duplicate. The CqPCR conditions were 95 °C for 10 min and 40 cycles at 95 °C for 15 s and 60 °C for 1 min. The qPCR product quality was monitored by a melting curve analysis (95 °C for 15 s and 60 °C for 1 min). qPCR was performed in an ABI 7300 real-time PCR cycler (Applied Biosystems, Waltham, MA, USA).

### 2.9. Macrophage Generation

Bone marrow cells were seeded in 12-well cell cluster plates (4 × 10^5^/1 mL) in an IMDM-based culture medium (see above), containing 10 ng/mL of recombinant murine M-CSF (Miltenyi Biotec). The culture medium was renewed on days 3 and 6 of culture. Derived macrophages (MACs) were used for experiments on day 7 of culture.

### 2.10. Phagocytosis Assay

Freshly isolated PMNs and bone marrow-derived MACs were reseeded into 96-well plates (10^5^/100 μL), and GFP-fluorescent AFC [28] were applied as indicated. Assays were set up in parallel at 4 °C and 37 °C, enabling the differentiation of mere adhesion (4 °C) and energy-dependent uptake (37 °C). After 3 h of incubation, the samples were washed twice (each 500 μL pre-cooled PBS) and incubated with CD11b plus either anti-Ly6G- (PMN) or anti-F4/80- (MAC) specific antibodies, as well as FVD eFluor 450, in order to assess the uptake of GFP-expressing conidia via flow cytometry. Figure S7 denotes the gating strategies.

### 2.11. ROS Detection

PMNs were seeded into 96-well plates (10^5^/100 µL), washed with PBS, and resuspended in 100 μL of 2 mM 2′−7′dichlorodihydrofluorescein (DCFDA, Alexis Biochemicals, Lausen, Switzerland) in PBS. After 20 min at 37 °C, the samples were washed and resuspended in 200 μL of PBS. Then, the samples were differentially treated in triplicate with 100 ng/mL of granulocyte/macrophage colony-stimulating factor (GM-CSF), 1 µg/mL of LPS, and 10^5^ AFC at 37 °C and 5% CO_2_. The mean fluorescence intensities were assessed (excitation: 485 nm; emission: 530 nm) for a period of 180 min, with readings taken at 15 min intervals using a SPARK multimode microplate reader (TECAN, Männedorf, Switzerland).

### 2.12. Apoptosis Detection

The PMNs were seeded in 96-well plates (10^5^/100 µL) and treated in parallel settings with LPS (1 μg/mL) in order to assess apoptosis under different conditions. After washing, Annexin V (FITC), anti-Ly6G antibody (PE) and FVD (eFluor 450) (all from Thermo Fisher, Waltham, MA, USA) were added to delineate early and late apoptotic cells. The gating strategy is illustrated in Figure S10.

### 2.13. Transcriptome Analysis

The NGS library preparation of the PMN-derived RNA was conducted using Illumina’s Stranded mRNA Prep Ligation Kit following Illumia’s standard protocol (Document 1000000124518 v02). All the libraries were prepared using 52.5 ng of RNA, followed by amplification by 11 PCR cycles. The PCR products were purified to remove the primer and adapter dimers. Then, the libraries were characterized using a High Sensitivity DNA chip on a 2100 Bioanalyzer (Agilent technologies, Santa Clara, CA, USA). The cDNA quantities were determined with a Qubit dsDNA HS Assay Kit, using a Qubit 4.0 Fluorometer (Life technologies, Carlsbad, CA, USA). All the samples were pooled at an equimolar ratio to enable at least 30 M reads/sample. Sequencing was performed on a NextSeq 2000 P3 in accordance with the recommendations of the manufacturer (Illumina, San Diego, CA, USA).

Afterwards, the reads were aligned using a STAR aligner (v2.7.3a; [39]) and the GRCm39 reference genome (settings: outStd SAM, outMultimapperOrder Random, outSAMattributes NH HI AS nM MD, outFilterMismatchNmax 999, outFilterMismatchNoverReadLmax 0.04). Primary alignments were assigned to exons using featurCounts (Subread software package v2.0.0; [40]) at the default parameters. Throughout the analysis, GENCODE mouse annotation release M26 was used. Uniquely mapped reads were subjected to differential expression analysis by applying Bioconductor (v3.14; [41]) and DESeq2 (v1.34.0; [42]). In this regard, genes characterized by a Benjamini–Hochberg-adjusted false discovery rate (FDR) below 0.1 were considered differentially expressed. Gene set enrichment analysis was conducted using the GSEA 4.2.3 software (default settings, gene set database: h.all.v7.5.1 [43,44]). An FDR q-value < 0.05 was considered statistically significant. Protein–protein interaction networks were analyzed using the STRING database (v12) [45]. A Venn diagram calculator (https://bioinformatics.psb.ugent.be/webtools/Venn/ (accessed on 14 September 2024)) was employed to calculate the number of genes expressed commonly and differentially by PMNs and MACs, respectively. The results of the PMN transcriptome analysis have been deposited in the GEO database (GSE280203).

### 2.14. Statistics

Statistical analysis was conducted with GraphPad Prism v9.5.1 (GraphPad Software Inc., San Diego, CA, USA). All results were expressed as the mean ± the standard error of the mean using three to seven mice in each experimental group for the spleen analysis, three mice/group for the IPA assay and RNA sequencing, six mice/group for the phagocytosis and apoptosis assays, and nine mice/group for the qPCR and ROS assays.

## 3. Results

### 3.1. KSRP Deficiency Resulted in the Increased Production of Proinflammatory Mediators After Stimulation

Sepsis is a condition that arises from an excessive response of the body to an infection. Under certain circumstances, MACs and PMNs, which typically protect the body from infection, can cause severe tissue damage, resulting in multiple organ failure, which is a principal clinical manifestation of sepsis [46]. We recently demonstrated that KSRP^−/−^ mice secrete elevated levels of proinflammatory mediators in an LPS-induced sepsis model, indicating that KSRP typically limits inflammatory responses [32]. We sought to determine whether this could yield advantages in an infection model. To evaluate the hyperactivity of KSRP-deficient immune cells toward a pathogen, spleen cells were incubated with *A. fumigatus*, which is known to cause fungal infection in the lungs of patients who are immunocompromised [25,47]. Here, especially alveolar MACs and PMNs, are primarily responsible for killing conidia [27,30].

**Figure 1:**
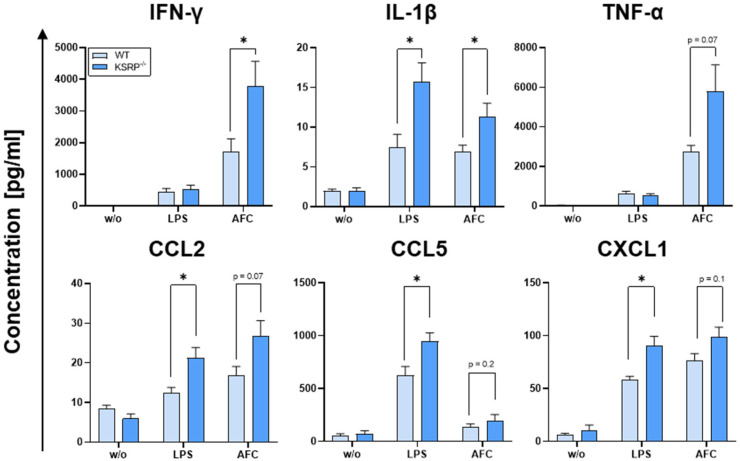
KSRP deficiency led to the higher production of proinflammatory mediators following LPS or AFC stimulation. Here, 10^6^ spleen cells were stimulated with 1 µg/mL of LPS or 1 × 10^6^ AFC for 16 h. Supernatants were collected, and the cytokine content was determined. Data denote the mean ± SEM of *n* = 4–7 analyses (* *p* < 0.05; two-tailed Student’s *t*-test).

Significant elevations in IFN-γ and IL-1β cytokine concentrations were observed following AFC stimulation, whereas TNF-α, CCL2, CCL5, and CXCL1 displayed trends toward significant elevations (Figure 1). It is noteworthy that IL-1β, CCL2, CCL5, and CXCL1 cytokine levels were also significantly elevated following LPS stimulation (Figure 1). In conclusion, these results show that KSRP limits the cytokine response to different TLR agonists (LPS-TLR-4 [48], AFC-TLR-2, and TLR-4 [25]), indicating that KSRP^−/−^ mice may exert a stronger anti-pathological immune reaction. Interestingly, PMNs and MACs exhibited no differences in their Dectin-1, TLR2, or TLR4 expression, even after stimulation with LPS or AFC (Figure S2). Additionally, with regard to activation markers, no genotype-specific differences could be detected after stimulation (Figure S3).

### 3.2. PMN Depletion in KSRP^−/−^ Mice Led to a Resistance to A. fumigatus Infection

**Figure 2:**
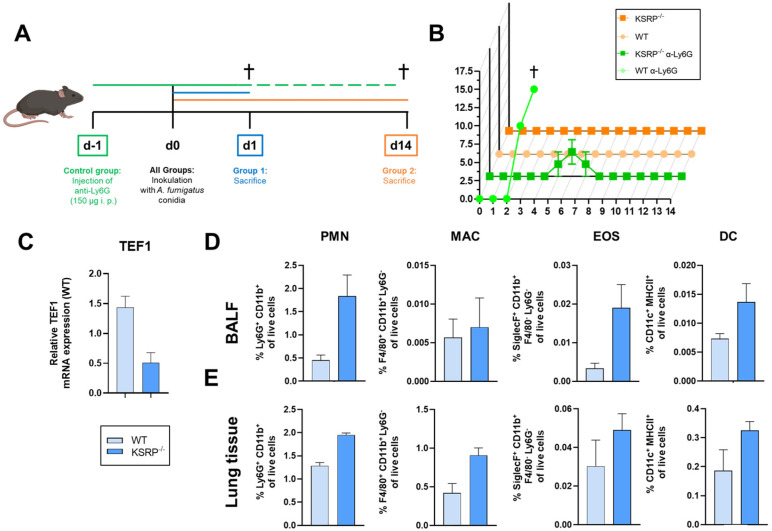
Depletion of PMNs and the inactivation of the KSRP gene led to resistance against *A. fumigatus* infection. (**A**) One day prior to fungal infection, the control mice were i.p. injected with an α-Ly6G antibody to achieve PMN depletion (green). 24 h after inoculation, the first group (blue) was sacrificed (†), whereas the second group (orange) was analyzed 14 days following inoculation with AFC (created with BioRender.com). (**B**) The clinical course of IPA was monitored for 2 weeks. (**C**) *TEF1* mRNA expression, as a marker for AFC [49], was assessed by qRT-PCR and normalized to *GAPDH* mRNA expression. The lungs of KSRP^−/−^ mice contained less AFC-specific mRNA compared to WT mice 1 day post-inoculation. (**D**) Flow cytometric analysis showed higher frequencies of PMNs in KSRP-deficient mice, whereas MACs, EOSs, and DCs showed no genotype-dependent differences in the BALF. (**E**) Flow cytometric analyses of lung tissue displayed higher frequencies of PMNs and MACs, whereas EOSs and DCs showed no genotype-dependent differences 1 day after inoculation. Data denote the mean ± SEM of three samples/group. Statistically significant differences are indicated (two-tailed Student’s *t*-test).

To evaluate this hypothesis, we initiated IPA, which is caused by the infection of individuals who are immunocompromised with *A. fumigatus* [35]. The eradication of AFC critically depends on PMN and MAC activity at an early stage [28,29,30]. Some animals were injected intraperitoneally (i.p.) with a Ly6G-specific antibody to deplete PMNs prior to intratracheal inoculation with *A. fumigatus* conidia (d0) to prove successful infection (Figure 2A). Surprisingly, in contrast to the WT control animals, the neutropenic KSRP^−/−^ mice showed only mild symptoms of disease (Figure 2B). All immunocompetent WT and KSRP^−/−^ mice survived infection, as monitored over 2 weeks (Figure 2B). Next, we investigated the initial innate immune response to infection. To this end, we performed a comprehensive analysis of the lung tissue, bronchoalveolar lavage fluid (BALF), and serum collected from the infected mice 1 day post-infection (Group 1). First, we quantified the fungal burden in the lung tissue of the WT and KSRP^−/−^ mice by measuring AFC-specific *elongation factor 1-alpha* (*TEF1*) mRNA expression, as previously described [49]. A quantitative polymerase chain reaction (qPCR) analysis demonstrated notably lower AFC levels in the KSRP^−/−^ mice relative to the WT mice (Figure 2C). Furthermore, we observed higher frequencies of PMNs in the BALF and of PMNs and MACs in the lung tissue of KSRP-deficient compared to WT mice (Figure 2D,E), whereas for eosinophilic granulocytes (EOSs) and dendritic cells (DCs) (Figure 2D,E), as well as for B cells, T cells, and NK cells (Figure S3A), no genotype-dependent alterations were apparent. Fourteen days following infection, the Group 2 mice were analyzed to assess the late onset of *A. fumigatus* infection. As previously stated, neutropenic KSRP^−/−^ mice demonstrated unexpected mild disease symptoms and were also analyzed 14 days post-inoculation. An analysis of lung lymph nodes revealed an increase in the numbers of T and B cells in anti-Ly6G-treated KSRP^−/−^ in comparison to WT mice (Figure S3C). These data suggest that infection can be combated more effectively by KSRP-deficient than by WT animals.

### 3.3. KSRP-Deficient PMNs Express Genes Involved in Inflammatory Responses to a Greater Extent

Due to the overall protective effect of KSRP deficiency against fungal infection associated with a higher influx of PMNs, we performed a transcriptome analysis of unstimulated and LPS-stimulated PMNs to elucidate the immunophenotypical differences (Figure S5A). The KSRP-deficient PMNs exhibited a reduced expression of 485 genes and an elevation in the abundance of 1070 genes in comparison to the corresponding control group (WT) (Figure S5B). Notably, GESA demonstrated that KSRP-deficient PMNs exhibited an attenuated expression of metabolism-relevant genes, including oxidative phosphorylation and glycolysis (Figure S5D).

**Figure 3:**
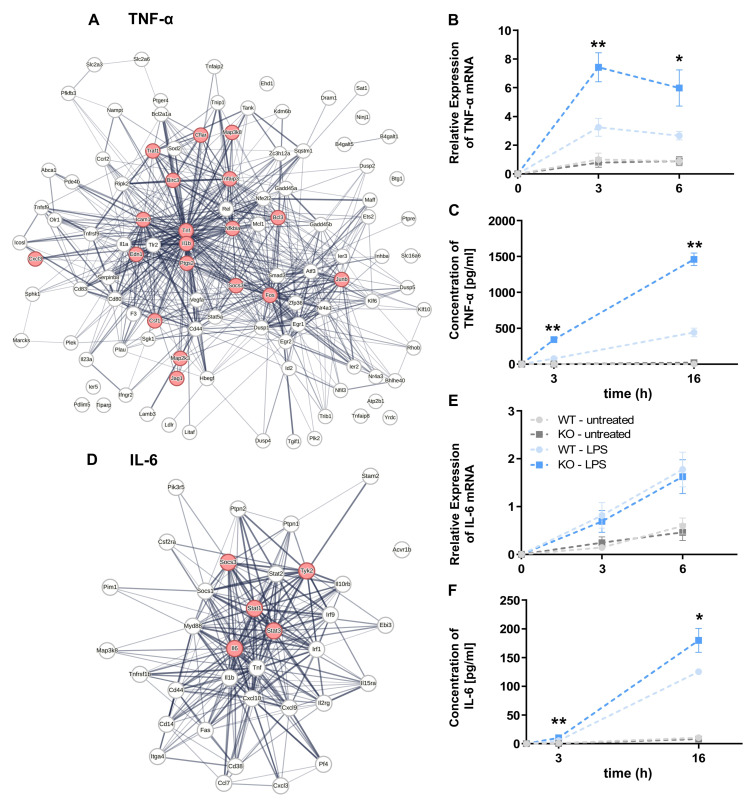
KSRP-deficient PMNs are characterized by the enhanced expression of genes involved in the inflammatory response upon stimulation. (**A**,**D**) TNF-α- and IL-6-related genes (red) were further analyzed for gene clusters using the STRING database. (**B**,**E**) The mRNA expression of TNF-α and IL-6 in PMNs stimulated with 1 µg/mL of LPS for different time periods (3 h and 6 h). Data denote the mean ± SEM of *n* = 9 (** *p* < 0.01, * *p* < 0.05; versus untreated WT cells; two-tailed Student’s *t*-test). (**C**,**F**) The protein expression of TNF-α and IL-6 in the supernatants of LPS-stimulated PMNs after different time periods (3 h and 16 h). Data denote the mean ± SEM of *n* = 6–9 samples/group (* *p* < 0.05, ** *p* < 0.01; two-tailed Student’s *t*-test).

In response to LPS stimulation, KSRP-deficient PMNs demonstrated the differential regulation of a total of 1716 genes, comprising 716 upregulated and 1000 downregulated genes (Figure S5C). GESA revealed the elevated expression of multiple genes in KSRP-deficient PMNs that are implicated in TNF-α and IL-6 signaling pathways (Figure S5E). In accordance with these findings, time-kinetics studies demonstrated that KSRP-deficient PMNs exhibited higher levels of *TNF-α* mRNA after 3 h following the initiation of LPS stimulation (Figure 3B). Additionally, KSRP-deficient PMNs exhibited elevated TNF-α levels at both the 3 and 16 h time points following LPS stimulation (Figure 3C). In contrast, no genotype-dependent differences were observed in *IL-6* mRNA levels (Figure 3E). However, KSRP-deficient PMNs exhibited increased IL-6 secretion following LPS treatment (Figure 3F), indicating that KSRP, besides affecting mRNA stability, may also attenuate mRNA translation in a targeted mRNA-specific manner, thereby impairing gene expression.

Since we recently reported that KSRP-deficient MACs produced proinflammatory cytokines at higher levels than WT MACs in response to LPS stimulation [32], we next investigated genotype-dependent transcriptional alterations between KSRP-deficient PMNs and MACs.

### 3.4. KSRP-Deficient PMNs and MACs Share the Upregualtion of Genes Involved in Pathogen Defense

**Figure 4:**
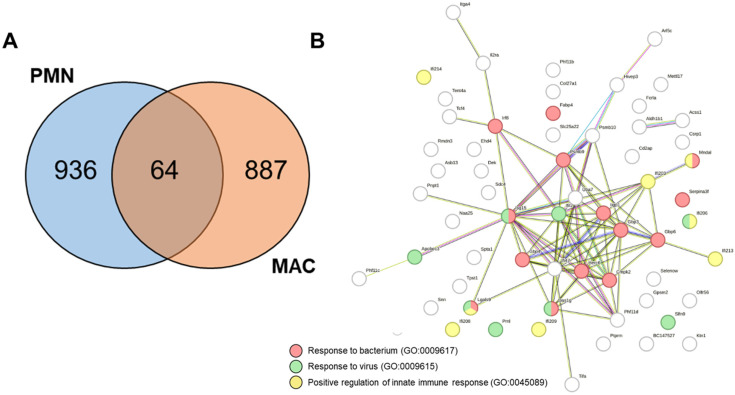
Upregulation of genes involved in pathogen defense in KSRP-deficient PMNs and MACs following LPS stimulation. (**A**) Using a Venn diagram calculator (https://bioinformatics.psb.ugent.be/webtools/Venn/ (accessed on 14 September 2024)), we calculated the same and different upregulated genes after LPS stimulation within PMNs and MACs. (**B**) The STRING database revealed that 64 equally upregulated genes in PMNs and BMDMs were interlinked and contributed to a positive defense regulation against pathogens and the activation of innate immunity.

To gain further insight into the similarities and differences in gene regulation in KSRP-deficient PMNs and MACs regarding their eminent role in pathogen defense, we conducted a comparative analysis of all upregulated genes following LPS stimulation in both cell types. Our findings revealed that 64 genes were upregulated in both cell types. Additionally, PMNs exhibited an upregulation of 936 genes, whereas MACs demonstrated an upregulation of 887 genes (Figure 4A).

A STRING database analysis revealed that many of the 64 genes congruently upregulated by KSRP-deficient PMNs and MACs were connected and contributed to the regulation of innate immune responses (5.4%) and anti-viral (2.3%) and anti-bacterial immune responses (1.7%) (Figure 4B).

The genes that exhibited differential upregulation between PMNs and MACs were also examined for gene clusters using the STRING database (Figure S6). This revealed that PMNs mainly upregulated genes associated with DNA replication (29.4%), DNA repair (18.8%), the cell cycle (14.5%), and cellular metabolic processes (6.7%) (Figure S6A). In contrast, MACs exhibited an upregulation of genes associated with anti-viral (22.4%), anti-bacterial (17.8%), and anti-fungal immune responses (13%) (Figure S6B).

### 3.5. KSRP Knockout Enhances PMN and MAC Effector Functions

#### 3.5.1. Phagocytosis

**Figure 5:**
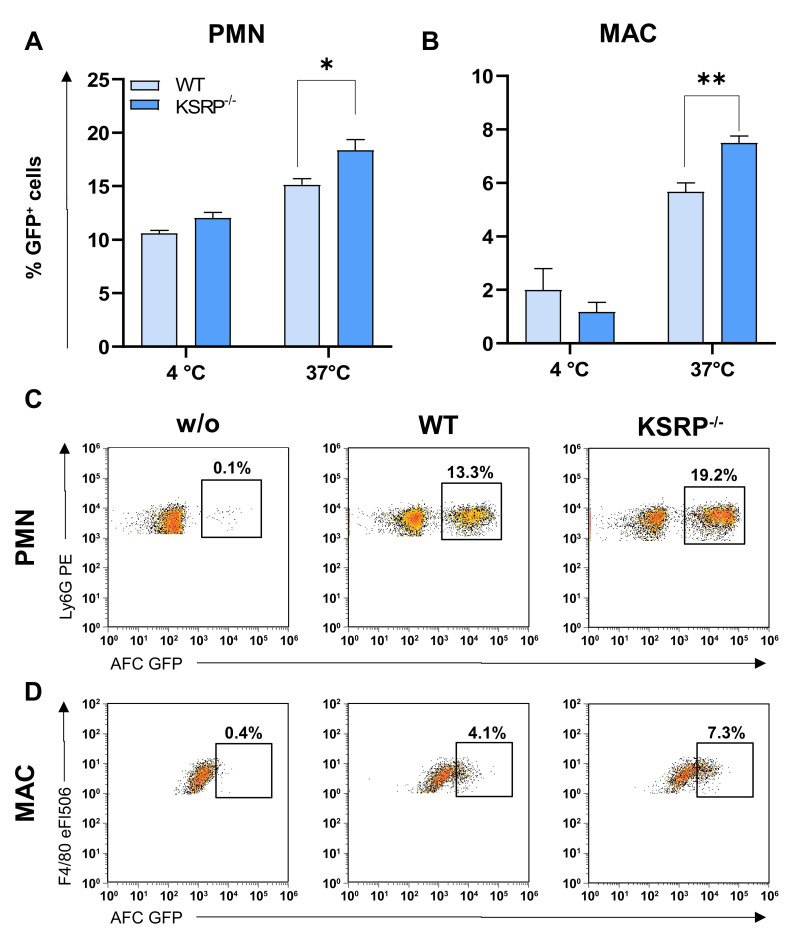
KSRP deficiency enhances PMN and MAC phagocytosis. PMNs were immunomagnetically isolated via Ly6G from the bone marrow of WT and KSRP^−/−^ mice, whereas BMDMs were differentiated from bone marrow with M-CSF for 7 days. (**A**,**B**) 1 × 10^5^ PMNs or BMDMs were cultured with serum-preincubated GFP-expressing AFC at 4 °C or 37 °C with the indicated ratios. This was performed both at 4 °C and 37 °C to distinguish between AFC adhesion and uptake, respectively. After 3 h, the frequencies of GFP-positive PMNs and MACs were assessed by flow cytometric analysis. Data denote the mean ± SEM of six samples/group (** *p* < 0.01, * *p* < 0.05; two-tailed Student’s *t*-test). Exemplary primary data showing the pronounced uptake of GFP-expressing AFC by KSRP-deficient PMNs (**C**) and MACs (**D**). The gating strategy is illustrated in Figure S7.

To analyze the potential effects of KSRP on the phagocytic activity of PMNs and MACs, as a major mechanism for pathogen killing, including *A. fumigatus* [50], we incubated GFP-labeled AFC with bone marrow-derived PMNs and day-7-differentiated MACs from WT and KSRP^−/−^ mice. We observed that PMNs (Figure 5A) and MACs (Figure 5B) from KSRP^−/−^ mice exhibited a significantly higher uptake of AFC at 37 °C three hours after incubation. Examples of flow cytometric data showing the elevated phagocytosis of GFP-fluorescent AFC by KSRP-deficient PMNs (Figure 5C) and MACs (Figure 5D) are presented below.

#### 3.5.2. ROS Production

**Figure 6:**
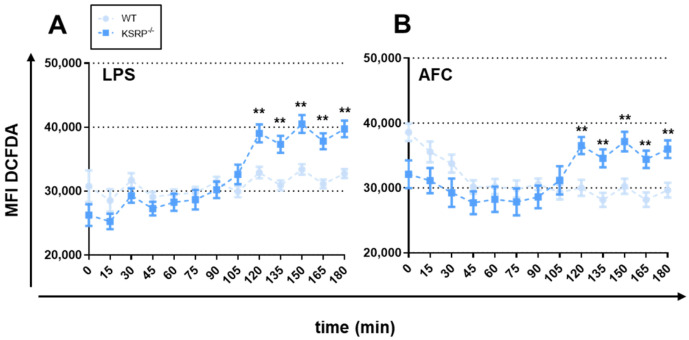
KSRP deficiency enhances ROS production by PMNs. Here, 10^5^ PMNs were stained with DCFDA, treated with 100 ng/mL of GM-CSF plus the indicated stimuli and 1 µg/mL of LPS (**A**) or 1 × 10^5^ AFC (**B**), and measured in 15 min intervals for 3 h. Data denote the mean ± SEM of nine samples/group (** *p* < 0.01; two-tailed Student’s *t*-test).

We then examined ROS generation as one further key effector mechanism in pathogen defense. Our findings show that PMNs from KSRP^−/−^ mice produced significantly higher levels of ROS following stimulation with LPS or AFC (Figure 6). PMNs treated with GM-CSF yielded higher ROS production only in WT cells, but it was still below the levels observed for KSRP-deficient PMNs after stimulation with LPS or AFC (Figure S8).

### 3.6. Stimulation of KSRP-Deficient PMNs Attenuates Apoptosis

**Figure 7:**
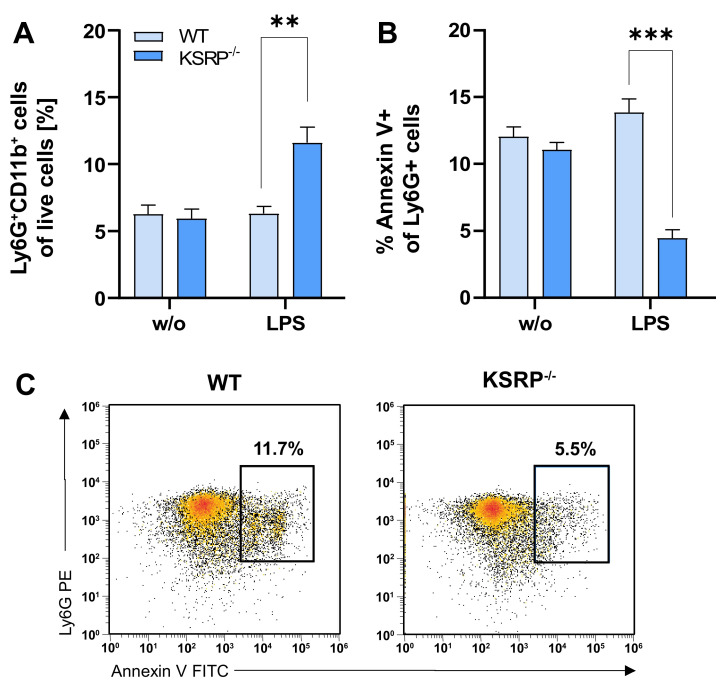
Stimulation with LPS leads to reduced apoptosis in the PMNs of KSRP^−/−^ mice. PMNs were immunomagnetically isolated via Ly6G from bone marrow. Here, 10^6^ PMNs were cultured without or with 1 µg/mL of LPS for 6 h. Afterwards, surface receptors were stained for flow cytometry analysis. (**A**) Stimulation leads to the increased frequency of PMNs in KSRP^−/−^ mice. Data denote the mean ± SEM of nine samples/group (** *p* < 0.01; two-tailed Student’s *t*-test). (**B**) An assessment of PMN apoptosis revealed a lower expression of the apoptosis marker Annexin V in stimulated KSRP-deficient PMNs. Shown are the mean ± SEM of *n* = 6 analyses (** *p* < 0.01, *** *p* < 0.001; two-tailed Student’s *t*-test). (**C**) Exemplary flow cytometry data showing attenuated apoptosis by PMNs from KSRP^−/−^ mice. The complete gating strategy is illustrated in Figure S9.

As the PMNs displayed an upregulation of genes associated with cell viability, we accordingly analyzed PMN viability at the basal state and upon treatment with LPS and AFC stimulation. Concerning the expression of early (CD62L or CD18) and late (CD80, CD86, MHCII) activation markers, no genotype-dependent differences were observed at either state of activation (Figure S9). Interestingly, the frequencies of PMNs in bone marrow under steady-state conditions were not altered by KSRP deficiency (w/o stimulation), whereas stimulation with LPS led to higher frequencies of PMNs in KSRP^−/−^ mice (Figure 7A). Similarly, lower levels of KSRP^−/−^ PMNs were apoptotic compared to WT PMNs, suggesting that KSRP may promote the apoptosis of PMNs (Figure 7B,C).

## 4. Discussion

Our previous research has demonstrated that the innate immune cells of KSRP^−/−^ mice generate elevated levels of proinflammatory mediators in an LPS-induced sepsis model, which indicates that KSRP plays an important role in limiting inflammatory responses [32]. In this study, we investigated whether an exacerbated innate immune response could offer benefits in an infection model. Therefore, we used *Aspergillus fumigatus*, which causes the most common fungal lung infection in patients who are immunocompromised [47]. In this context, the innate immune system, particularly PMNs [35] and MACs [25,30], serves as the primary defense mechanism responsible for clearing conidia and preventing the growth of AFC [27].

Previous studies have indicated that IFN-γ [51,52], IL-1β [53], TNF-α [52], CCL2 [54], CCL5 [55], and CXCL1 [56] exert a pivotal role in the host defense against IPA. When incubated with AFC, splenic immune cells lacking KSRP exhibit the augmented production of various anti-fungal cytokines, including IL-1β, IFN-γ, CCL2, and TNF-α, suggesting that KSRP-deficient mice may demonstrate a more robust anti-fungal immune response.

Consistent with this, a comparative analysis of the upregulated genes in KSRP-deficient PMNs and MACs after LPS stimulation demonstrated 64 shared upregulated genes. Using the STRING database, we revealed that these contribute to the pathogen defense response, suggesting elevated pathogen defense activity in the absence of KSRP.

As phagocytosis is one key effector mechanism by which PMNs and MACs clear pathogen infections [19], we analyzed the effect of KSRP deficiency in this context. PMNs and MACs derived from KSRP^−/−^ mice exhibited increased phagocytic activity against GFP-labeled AFC, indicating that PMNs and MACs may prevent the growth of AFC in vivo.

To assess this hypothesis, we initiated IPA in WT and KSRP^−/−^ mice through AFC inoculation. One day after infection, we observed that the KSRP-deficient mice exhibited a lower fungal load in the lungs of KSRP-deficient animals, which was accompanied by elevated frequencies of PMNs in the BALF and of PMNs and MACs in lung tissue, suggesting a faster clearance of AFC by these immune cells. In addition, we recently demonstrated that KSRP attenuates the migration of PMNs toward CXCL1 [57], the most crucial chemoattractant for PMNs in the context of infection [58].

It was unexpected that the PMN-deficient KSRP^−/−^ mice would survive inoculation with AFC, whereas the WT control group died within the first 3 days. These findings indicate that additional factors may be responsible for the suppression of AFC to hyphae. In this regard, our findings of the elevated phagocytic activity of KSRP-deficient MACs in vitro, the enhanced MAC numbers in the lungs of KSRP-deficient AFC-infected mice, and the MAC-specific upregulation of gene sets involved in pathogen defense suggest that these immune cell populations may compensate for the loss of antibody-mediated PMNs.

The adaptive immune system is indispensable for controlling and eliminating infection, particularly in individuals who are immunocompromised, such as PMN-deficient mice. It is noteworthy that, 14 days post-inoculation, an increase in T and B cell frequencies was observed in the lung-associated lymph nodes of KSRP-deficient mice. Further studies are necessary to delineate whether this difference is due to the KSRP-dependent intrinsically stronger immune activity of the adaptive immune system.

Furthermore, our findings revealed that KSRP deficiency resulted in elevated ROS production in PMNs. The data presented here suggest that KSRP may influence the regulation of oxidative stress-induced gene expression. It has been demonstrated that multiple targets of KSRP encode mRNAs that are regulated by oxidative stress [59,60]. KSRP activity may be regulated by protein kinases, e.g., p38 or PKB/Akt, that phosphorylate and thereby inhibit KSRP in its ARE-mediated decay of target mRNAs [61]. It is noteworthy that TTP, which also exhibits a binding affinity for numerous ROS-regulated mRNAs (e.g., *c-fos*, *GM-CSF*, and *COX-2*), was observed to be present at enhanced levels in the skeletal muscle and liver of mice fed a high-glucose diet, triggering inflammation and oxidative stress [61].

Moreover, the transcriptome analysis of LPS-stimulated PMNs revealed an increased expression of genes involved in IL-6 and TNF-α signaling in KSRP-deficient PMNs, which are also involved in IPA clearance. These findings were confirmed by time-kinetics studies, revealing elevated mRNA and protein levels for TNF-α, although there were no genotype-dependent differences in *IL-6* at the RNA level. However, KSRP-deficient PMNs displayed higher IL-6 concentrations after 3 h and 16 h. Similarly, Dhamija and colleagues demonstrated that KSRP engaged the AREs of *IL-6* mRNA, thereby mediating translational inhibition [5], suggesting that KSRP acts not at a transcriptional but at translational level.

Quite a number of genes enhanced in expression by stimulated KSRP-deficient PMNs are involved in the signaling pathways that regulate DNA repair cell cycle progression. Consistent with this, KSRP^−/−^ PMNs were characterized by attenuated apoptosis, suggesting that KSRP deficiency prolongs cell survival, leading to an association between elevated effector mechanisms and pronounced pathogen defense. Ebner and co-workers determined that TTP-deficient PMNs express higher levels of the TTP-target *Mcl1* mRNA, which codes for an anti-apoptotic factor particularly relevant for neutrophils [62]. Although TTP deficiency reduces PMN apoptosis, the PMN homeostasis under steady-state conditions is not impaired, implying that TTP regulates PMN survival upon infection but not during homeostatic differentiation or circulation [62].

Taken together, our results suggest that KSRP is a critical negative regulator of anti-pathogen activity in PMNs and MACs. The inhibition of KSRP in PMNs and MACs has the potential to enhance the body’s defense against infection in patients with limited innate immunity. This may be achieved, for instance, through the use of RNA interference.

## Figures and Tables

**Table 1:** Antibodies used for the investigations.

Surface Marker	Dye	Clone
CD86	FITC	GL-1
	eFluor450	
CD80	PerCP-eFl710	16-10A1
CD11c	APC	N418
MHC II	Brilliant Violet 421	M5/114.15.2
	eFluor 450	
	FITC	
CD11b	Super Bright 600	M1/70
Ly6G	PE	1A8
	PE-eFluor 610	
	APC	RB6-8C5
CD62L	PE-Cyanine 7	MEL-14
F4/80	eFluor 506	BM8
	APC	
CD3	eFluor 506	145-2C11
CD19	Super Bright 702	1D3
	PerCP-Cyanine 5.5	
NK1.1	PE	PK136
SiglecF	Brilliant Violet421	E50-2440
CD11c	Brilliant Violet421	N418
	APC	
TLR4	PE	MTS510
TLR2	PE	CB225
Dectin-1	APC	Bg1fpj
Annexin V	FITC	-

## Data Availability

The original contributions presented in this study are included in the article/Supplementary Material. Further inquiries can be directed to the corresponding author.

## Supplementary Materials

The following supporting information can be downloaded at: https://www.mdpi.com/article/10.3390/cells13242040/s1, Figure S1: Graphical representation of investigated cell types of the different murine organs, Figure S2. No genotype-dependent differences of TLR expression on splenocytes obtained from WT and KSRP^−/−^, Figure S3. Stimulation of splenic PMN and MAC from WT and KSRP^−/−^ mice has no genotype-dependent impact on surface activation marker expression, Figure S4. Flow cytometric analysis of IPA-treated mice, Figure S5. RNA-Sequencing results of WT and KSRP^−/−^ PMN, Figure S6. Genes showing differential upregulation between PMN and MAC were further analyzed for gene clusters using the STRING database, Figure S7. Gating strategy to evaluated phagocytic uptake of GFP-labeled AFC for PMN (A) and MAC (B), Figure S8. PMN from KSRP^−/−^ mice produced significantly higher levels of ROS following stimulation, Figure S9. Stimulation of bone-marrow-derived PMN from WT and KSRP^−/−^ mice has no genotype-dependent impact on (early) surface activation marker expression, Figure S10. Gating strategy to evaluated apoptosis levels of PMN in WT and KSRP^−/−^ mice.

## References

[1] Briata P., Chen C.Y., Ramos A., Gherzi R. (2013). Functional and molecular insights into KSRP function in mRNA decay. Biochim. Biophys. Acta.

[2] Linker K., Pautz A., Fechir M., Hubrich T., Greeve J., Kleinert H. (2005). Involvement of KSRP in the post-transcriptional regulation of human iNOS expression-complex interplay of KSRP with TTP and HuR. Nucleic Acids Res..

[3] Schmidtke L., Meineck M., Saurin S., Otten S., Gather F., Schrick K., Käfer R., Roth W., Kleinert H., Weinmann-Menke J. (2021). Knockout of the KH-Type Splicing Regulatory Protein Drives Glomerulonephritis in MRL-Fas(lpr) Mice. Cells.

[4] Gherzi R., Chen C.Y., Ramos A., Briata P. (2014). KSRP controls pleiotropic cellular functions. Semin. Cell Dev. Biol..

[5] Dhamija S., Kuehne N., Winzen R., Doerrie A., Dittrich-Breiholz O., Thakur B.K., Kracht M., Holtmann H. (2011). Interleukin-1 activates synthesis of interleukin-6 by interfering with a KH-type splicing regulatory protein (KSRP)-dependent translational silencing mechanism. J. Biol. Chem..

[6] Davis-Smyth T., Duncan R.C., Zheng T., Michelotti G., Levens D. (1996). The far upstream element-binding proteins comprise an ancient family of single-strand DNA-binding transactivators. J. Biol. Chem..

[7] Min H., Turck C.W., Nikolic J.M., Black D.L. (1997). A new regulatory protein, KSRP, mediates exon inclusion through an intronic splicing enhancer. Genes Dev..

[8] Gulei D., Raduly L., Broseghini E., Ferracin M., Berindan-Neagoe I. (2019). The extensive role of miR-155 in malignant and non-malignant diseases. Mol. Aspects Med..

[9] Nicastro G., García-Mayoral M.F., Hollingworth D., Kelly G., Martin S.R., Briata P., Gherzi R., Ramos A. (2012). Noncanonical G recognition mediates KSRP regulation of let-7 biogenesis. Nat. Struct. Mol. Biol..

[10] Yazarlou F., Kadkhoda S., Ghafouri-Fard S. (2021). Emerging role of let-7 family in the pathogenesis of hematological malignancies. Biomed. Pharmacother..

[11] Deng B., Tang X., Wang Y. (2021). Role of microRNA-129 in cancer and non-cancerous diseases (Review). Exp. Ther. Med..

[12] Bhattacharyya S., Kumar P., Tsuchiya M., Bhattacharyya A., Biswas R. (2013). Regulation of miR-155 biogenesis in cystic fibrosis lung epithelial cells: Antagonistic role of two mRNA-destabilizing proteins, KSRP and TTP. Biochem. Biophys. Res. Commun..

[13] Ruggiero T., Trabucchi M., De Santa F., Zupo S., Harfe B.D., McManus M.T., Rosenfeld M.G., Briata P., Gherzi R. (2009). LPS induces KH-type splicing regulatory protein-dependent processing of microRNA-155 precursors in macrophages. FASEB J..

[14] Chan L., Karimi N., Morovati S., Alizadeh K., Kakish J.E., Vanderkamp S., Fazel F., Napoleoni C., Alizadeh K., Mehrani Y. (2021). The Roles of Neutrophils in Cytokine Storms. Viruses.

[15] Wei Y., Yang L., Pandeya A., Cui J., Zhang Y., Li Z. (2022). Pyroptosis-Induced Inflammation and Tissue Damage. J. Mol. Biol..

[16] Minaga K., Watanabe T., Hara A., Yoshikawa T., Kamata K., Kudo M. (2021). Plasmacytoid Dendritic Cells as a New Therapeutic Target for Autoimmune Pancreatitis and IgG4-Related Disease. Front. Immunol..

[17] Kafasla P., Skliris A., Kontoyiannis D.L. (2014). Post-transcriptional coordination of immunological responses by RNA-binding proteins. Nat. Immunol..

[18] Kourtzelis I., Hajishengallis G., Chavakis T. (2020). Phagocytosis of Apoptotic Cells in Resolution of Inflammation. Front. Immunol..

[19] Lee W.L., Harrison R.E., Grinstein S. (2003). Phagocytosis by neutrophils. Microbes Infect..

[20] Greene J.T., Brian B.F.t., Senevirathne S.E., Freedman T.S. (2021). Regulation of myeloid-cell activation. Curr. Opin. Immunol..

[21] Sokol C.L., Luster A.D. (2015). The chemokine system in innate immunity. Cold Spring Harb. Perspect. Biol..

[22] Lendeckel U., Venz S., Wolke C. (2022). Macrophages: Shapes and functions. Chemtexts.

[23] Burn G.L., Foti A., Marsman G., Patel D.F., Zychlinsky A. (2021). The Neutrophil. Immunity.

[24] Wang K., Espinosa V., Rivera A. (2023). Commander-in-chief: Monocytes rally the troops for defense against aspergillosis. Curr. Opin. Immunol..

[25] Meier A., Kirschning C.J., Nikolaus T., Wagner H., Heesemann J., Ebel F. (2003). Toll-like receptor (TLR) 2 and TLR4 are essential for Aspergillus-induced activation of murine macrophages. Cell Microbiol..

[26] Feldmesser M. (2006). Role of neutrophils in invasive aspergillosis. Infect. Immun..

[27] Braedel S., Radsak M., Einsele H., Latgé J.P., Michan A., Loeffler J., Haddad Z., Grigoleit U., Schild H., Hebart H. (2004). *Aspergillus fumigatus* antigens activate innate immune cells via toll-like receptors 2 and 4. Br. J. Haematol..

[28] Teschner D., Cholaszczyńska A., Ries F., Beckert H., Theobald M., Grabbe S., Radsak M., Bros M. (2019). CD11b Regulates Fungal Outgrowth but Not Neutrophil Recruitment in a Mouse Model of Invasive Pulmonary Aspergillosis. Front. Immunol..

[29] Mircescu M.M., Lipuma L., van Rooijen N., Pamer E.G., Hohl T.M. (2009). Essential role for neutrophils but not alveolar macrophages at early time points following Aspergillus fumigatus infection. J. Infect. Dis..

[30] Luther K., Torosantucci A., Brakhage A.A., Heesemann J., Ebel F. (2007). Phagocytosis of Aspergillus fumigatus conidia by murine macrophages involves recognition by the dectin-1 beta-glucan receptor and Toll-like receptor 2. Cell. Microbiol..

[31] Braem S.G., Rooijakkers S.H., van Kessel K.P., de Cock H., Wösten H.A., van Strijp J.A., Haas P.J. (2015). Effective Neutrophil Phagocytosis of Aspergillus fumigatus Is Mediated by Classical Pathway Complement Activation. J. Innate Immun..

[32] Bolduan V., Palzer K.A., Hieber C., Schunke J., Fichter M., Schneider P., Grabbe S., Pautz A., Bros M. (2024). The mRNA-Binding Protein KSRP Limits the Inflammatory Response of Macrophages. Int. J. Mol. Sci..

[33] Lin W.J., Zheng X., Lin C.C., Tsao J., Zhu X., Cody J.J., Coleman J.M., Gherzi R., Luo M., Townes T.M. (2011). Posttranscriptional control of type I interferon genes by KSRP in the innate immune response against viral infection. Mol. Cell. Biol..

[34] Lother J., Breitschopf T., Krappmann S., Morton C.O., Bouzani M., Kurzai O., Gunzer M., Hasenberg M., Einsele H., Loeffler J. (2014). Human dendritic cell subsets display distinct interactions with the pathogenic mould Aspergillus fumigatus. Int. J. Med. Microbiol..

[35] Prüfer S., Weber M., Stein P., Bosmann M., Stassen M., Kreft A., Schild H., Radsak M.P. (2014). Oxidative burst and neutrophil elastase contribute to clearance of Aspergillus fumigatus pneumonia in mice. Immunobiology.

[36] Alflen A., Prüfer S., Ebner K., Reuter S., Aranda Lopez P., Scharrer I., Banno F., Stassen M., Schild H., Jurk K. (2017). ADAMTS-13 regulates neutrophil recruitment in a mouse model of invasive pulmonary aspergillosis. Sci. Rep..

[37] Hasenberg A., Hasenberg M., Männ L., Neumann F., Borkenstein L., Stecher M., Kraus A., Engel D.R., Klingberg A., Seddigh P. (2015). Catchup: A mouse model for imaging-based tracking and modulation of neutrophil granulocytes. Nat. Methods.

[38] Chomczynski P., Sacchi N. (1987). Single-step method of RNA isolation by acid guanidinium thiocyanate-phenol-chloroform extraction. Anal. Biochem..

[39] Dobin A., Davis C.A., Schlesinger F., Drenkow J., Zaleski C., Jha S., Batut P., Chaisson M., Gingeras T.R. (2013). STAR: Ultrafast universal RNA-seq aligner. Bioinformatics.

[40] Liao Y., Smyth G.K., Shi W. (2014). featureCounts: An efficient general purpose program for assigning sequence reads to genomic features. Bioinformatics.

[41] Gentleman R.C., Carey V.J., Bates D.M., Bolstad B., Dettling M., Dudoit S., Ellis B., Gautier L., Ge Y., Gentry J. (2004). Bioconductor: Open software development for computational biology and bioinformatics. Genome Biol..

[42] Love M.I., Huber W., Anders S. (2014). Moderated estimation of fold change and dispersion for RNA-seq data with DESeq2. Genome Biol..

[43] Mootha V.K., Lindgren C.M., Eriksson K.F., Subramanian A., Sihag S., Lehar J., Puigserver P., Carlsson E., Ridderstråle M., Laurila E. (2003). PGC-1alpha-responsive genes involved in oxidative phosphorylation are coordinately downregulated in human diabetes. Nat. Genet..

[44] Subramanian A., Tamayo P., Mootha V.K., Mukherjee S., Ebert B.L., Gillette M.A., Paulovich A., Pomeroy S.L., Golub T.R., Lander E.S. (2005). Gene set enrichment analysis: A knowledge-based approach for interpreting genome-wide expression profiles. Proc. Natl. Acad. Sci. USA.

[45] Szklarczyk D., Kirsch R., Koutrouli M., Nastou K., Mehryary F., Hachilif R., Gable A.L., Fang T., Doncheva N.T., Pyysalo S. (2023). The STRING database in 2023: Protein-protein association networks and functional enrichment analyses for any sequenced genome of interest. Nucleic Acids Res..

[46] Cohen J. (2002). The immunopathogenesis of sepsis. Nature.

[47] Lamoth F., Calandra T. (2022). Pulmonary aspergillosis: Diagnosis and treatment. Eur. Respir. Rev..

[48] Park B.S., Lee J.-O. (2013). Recognition of lipopolysaccharide pattern by TLR4 complexes. Exp. Mol. Med..

[49] Gravelat F.N., Doedt T., Chiang L.Y., Liu H., Filler S.G., Patterson T.F., Sheppard D.C. (2008). In vivo analysis of Aspergillus fumigatus developmental gene expression determined by real-time reverse transcription-PCR. Infect. Immun..

[50] Philippe B., Ibrahim-Granet O., Prévost M.C., Gougerot-Pocidalo M.A., Sanchez Perez M., Van der Meeren A., Latgé J.P. (2003). Killing of Aspergillus fumigatus by alveolar macrophages is mediated by reactive oxidant intermediates. Infect. Immun..

[51] Centeno-Lima S., Silveira H., Casimiro C., Aguiar P., do Rosário V.E. (2002). Kinetics of cytokine expression in mice with invasive aspergillosis: Lethal infection and protection. FEMS Immunol. Med. Microbiol..

[52] Nagai H., Guo J., Choi H., Kurup V. (1995). Interferon-gamma and tumor necrosis factor-alpha protect mice from invasive aspergillosis. J. Infect. Dis..

[53] Ralph B.A., Lehoux M., Ostapska H., Snarr B.D., Caffrey-Carr A.K., Fraser R., Saleh M., Obar J.J., Qureshi S.T., Sheppard D.C. (2021). The IL-1 Receptor Is Required to Maintain Neutrophil Viability and Function During Aspergillus fumigatus Airway Infection. Front. Immunol..

[54] Morrison B.E., Park S.J., Mooney J.M., Mehrad B. (2003). Chemokine-mediated recruitment of NK cells is a critical host defense mechanism in invasive aspergillosis. J. Clin. Investig..

[55] Schuh J.M., Blease K., Brühl H., Mack M., Hogaboam C.M. (2003). Intrapulmonary targeting of RANTES/CCL5-responsive cells prevents chronic fungal asthma. Eur. J. Immunol..

[56] Mehrad B., Wiekowski M., Morrison B.E., Chen S.C., Coronel E.C., Manfra D.J., Lira S.A. (2002). Transient lung-specific expression of the chemokine KC improves outcome in invasive aspergillosis. Am. J. Respir. Crit. Care Med..

[57] Palzer K.A., Bolduan V., Käfer R., Kleinert H., Bros M., Pautz A. (2022). The Role of KH-Type Splicing Regulatory Protein (KSRP) for Immune Functions and Tumorigenesis. Cells.

[58] Sawant K.V., Poluri K.M., Dutta A.K., Sepuru K.M., Troshkina A., Garofalo R.P., Rajarathnam K. (2016). Chemokine CXCL1 mediated neutrophil recruitment: Role of glycosaminoglycan interactions. Sci. Rep..

[59] Chen C.Y., Gherzi R., Ong S.E., Chan E.L., Raijmakers R., Pruijn G.J., Stoecklin G., Moroni C., Mann M., Karin M. (2001). AU binding proteins recruit the exosome to degrade ARE-containing mRNAs. Cell.

[60] Gherzi R., Lee K.-Y., Briata P., Wegmüller D., Moroni C., Karin M., Chen C.-Y. (2004). A KH domain RNA binding protein, KSRP, promotes ARE-directed mRNA turnover by recruiting the degradation machinery. Mol. Cell.

[61] Abdelmohsen K., Kuwano Y., Kim H.H., Gorospe M. (2008). Posttranscriptional gene regulation by RNA-binding proteins during oxidative stress: Implications for cellular senescence. Biol. Chem..

[62] Ebner F., Sedlyarov V., Tasciyan S., Ivin M., Kratochvill F., Gratz N., Kenner L., Villunger A., Sixt M., Kovarik P. (2017). The RNA-binding protein tristetraprolin schedules apoptosis of pathogen-engaged neutrophils during bacterial infection. J. Clin. Investig..

