# Supplementary material for: KSRP Deficiency Attenuates the Course of Pulmonary Aspergillosis and Is Associated with the Elevated Pathogen-Killing Activity of Innate Myeloid Immune Cells
Source: Cells. 2024 Dec 10;13(24):2040. doi: 10.3390/cells13242040 (PMC11674352; doi:10.3390/cells13242040)
Supplement: Supplementary file 1 [file cells-13-02040-s001.zip › cells-3256435-supplementary.pdf]

*Supplementary Material*

# KSRP deficiency attenuates the course of pulmonary aspergillosis, associated with elevated pathogen killing activity of innate myeloid immune cells

**Vanessa Bolduan[1], Kim-Alicia Palzer[2], Frederic Ries[3], Nora Busch[1], Andrea Pautz[2], Matthias Bros[1],***

[1] Department of Dermatology, University Medical Center of the Johannes Gutenberg University Mainz, Mainz, Germany
[2] Department of Pharmacology, University Medical Center of the Johannes Gutenberg University Mainz, Mainz, Germany
[3] Department of Hematology and Medical Oncology, University Medical Center of the Johannes Gutenberg University Mainz, Mainz, Germany
* Correspondence: mbros@uni-mainz.de

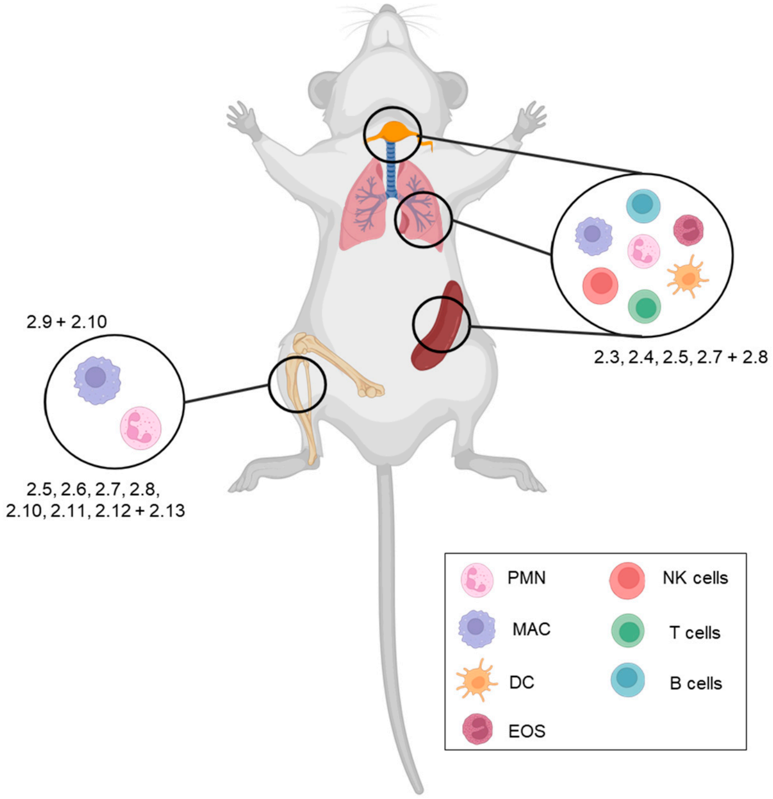


**Figure S1.** Graphical representation of investigated cell types of the different murine organs, as well as the references of the methods performed with them. PMN = polymorphonuclear neutrophils, MAC = macrophages, DC = dedritic cells, EOS = eosinophilic granulocytes.

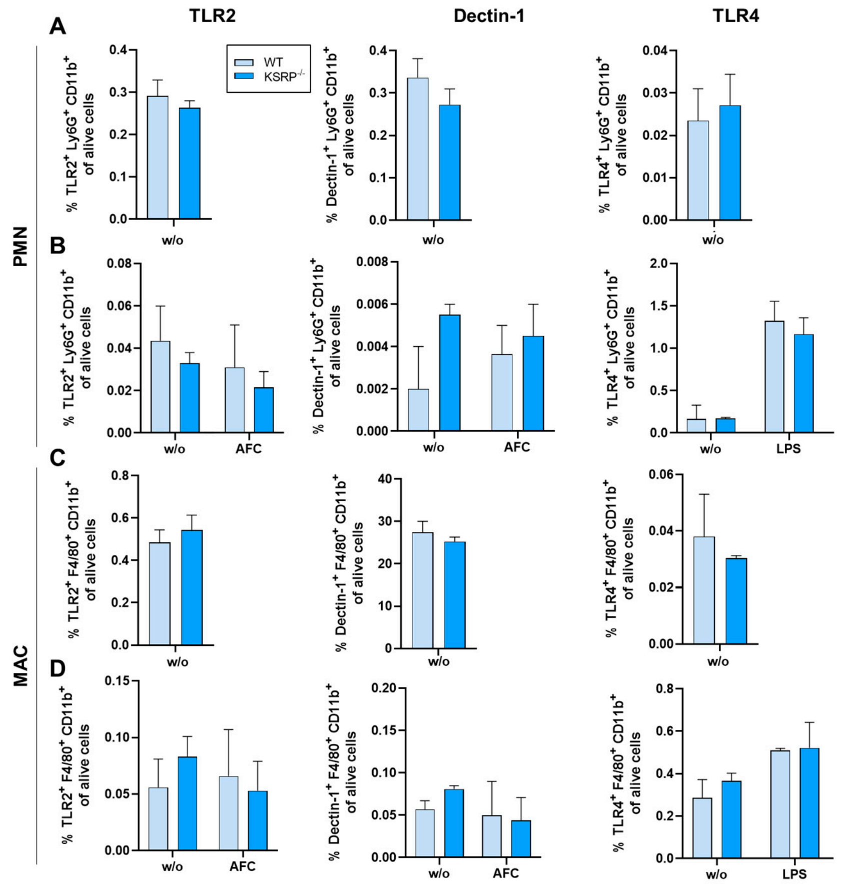


Figure S2. No genotype-dependent differences of TLR expression on splenocytes obtained from WT and KSRP$^{-/-}$. Freshly isolated spleen cells were stained for TLR2, Dectin-1 and TLR4 expression, with regard to PMN (A) or MAC (C). (B + D) Spleenocytes were treated for 16 h without (w/o) stimulation, 1 µg/ml LPS or 2x10$^6$ AFC. Flow cytometric analyses concerning TLR2, Dectin-1 and TLR4 expression on PMN (B) and MAC (D) revealed no genotype-dependent differences.

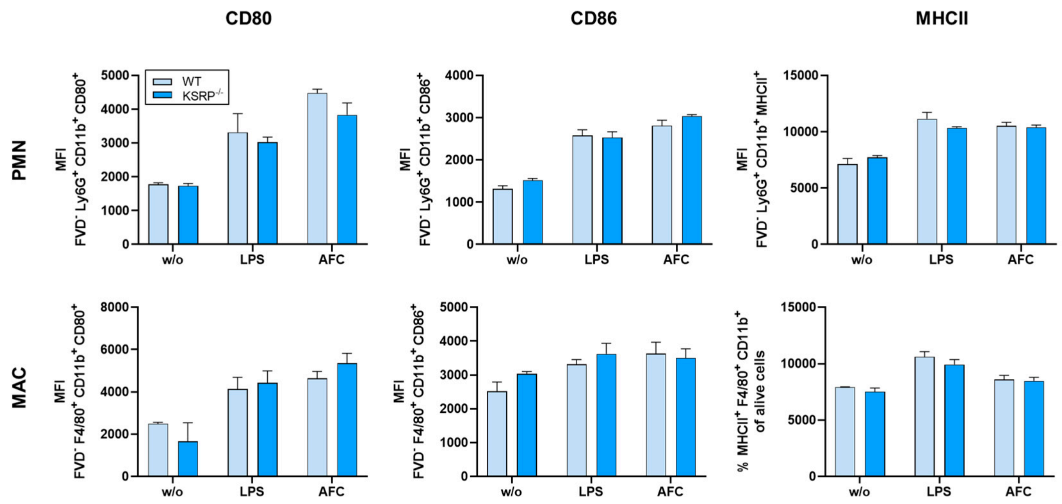


**Figure S3.** Stimulation of splenic PMN and MAC from WT and KSRP$^{-/-}$ mice has no genotype-dependent impact on surface activation marker expression.

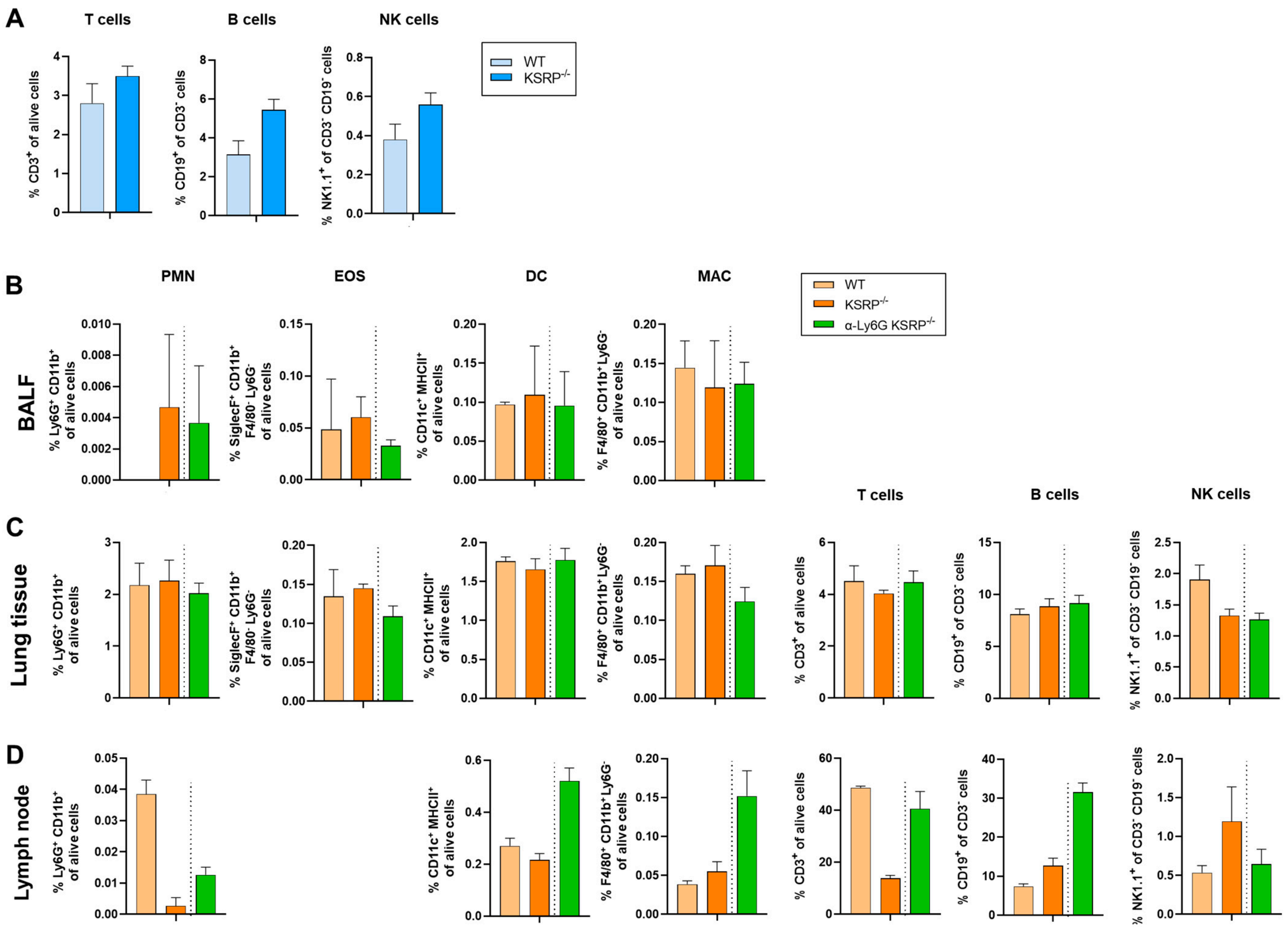


Figure S4. Flow cytometric analysis of IPA-treated mice. (A) No genotype-dependent differences were detectable regarding T, B and NK cells one day post inoculation. (B) Flow cytometric analyses of BALF (B), lung tissue (C) and the lung-associated lymph node (D) revealed significant levels of DC and T and B cells in the lymph node. PMN, EOS, DC and MAC displayed no genotype-depended differences 14 days after inoculation.

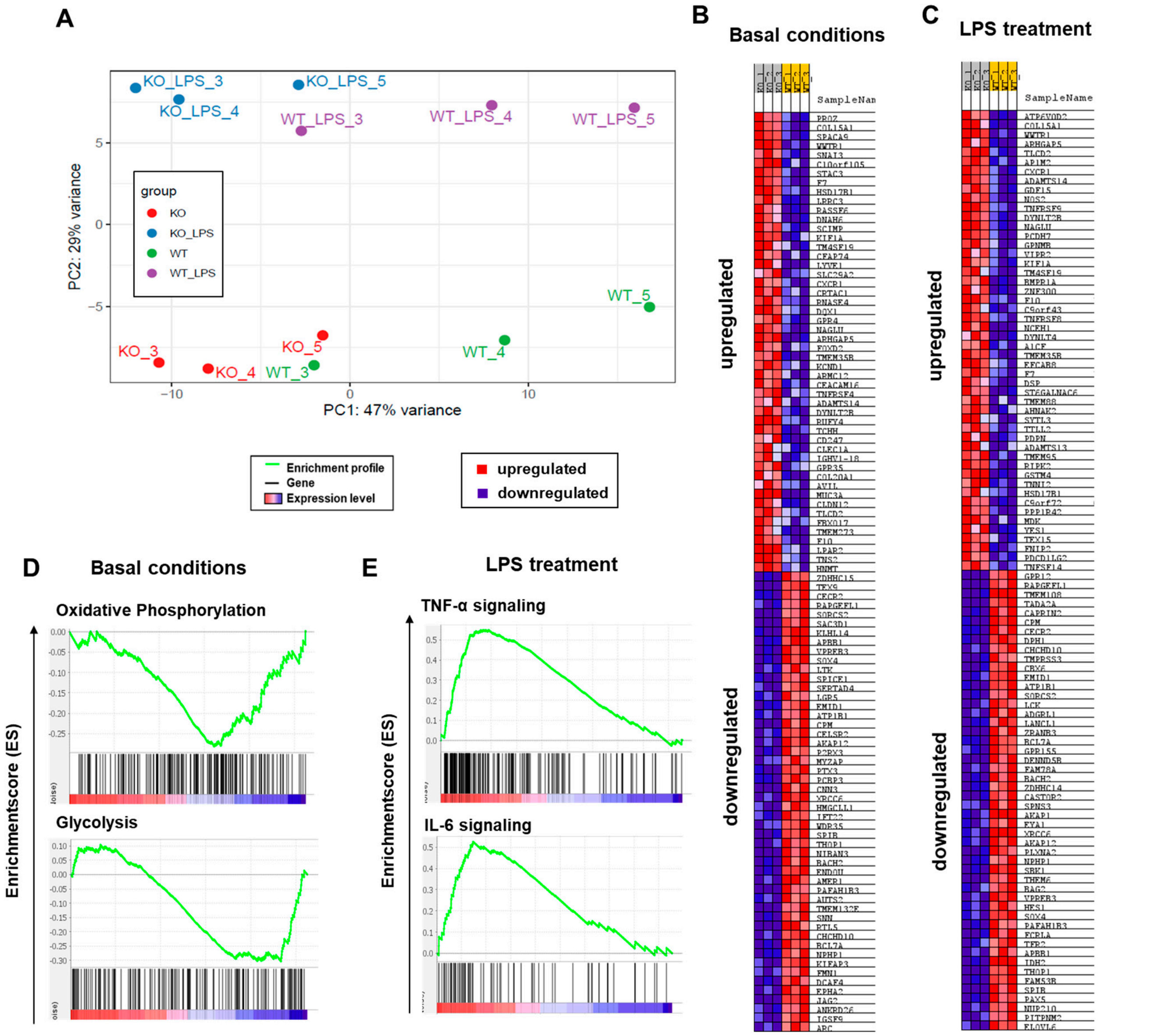


Figure S5. RNA-Sequencing results of WT and KSRP$^{-/-}$ PMN. (A) PCA plots showing different groups used for RNA-Sequencing. (B) Top 50 up- and downregulated genes in KSRP$^{-/-}$ PMN under basal conditions and (C) after stimulation with 1 μg/μl LPS. (D) GESA revealed that KSRP-deficient PMNs showed without stimulation a downregulation of genes associated with metabolic pathways, such as oxidative phosphorylation and glycolysis. (BH-adjusted $p < 0.05$). (E) . Gene set enrichment plots of TNF-α and IL-6 signaling pathways are significantly upregulated in PMN of KSRP$^{-/-}$ mice (BH-adjusted $p < 0.05$).

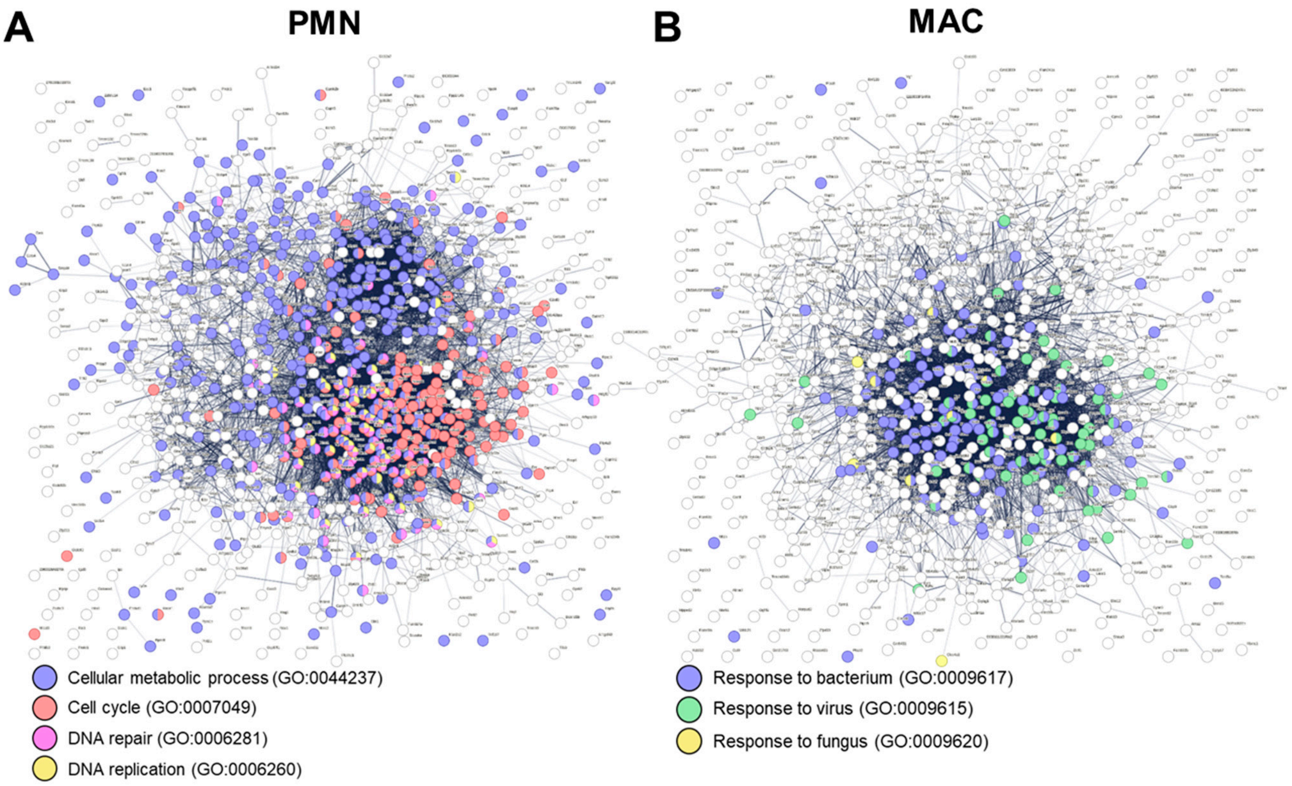


Figure S6. Genes showing differential upregulation between PMN and MAC were further analyzed for gene clusters using the STRING database. This analysis revealed that PMN (A) predominantly upregulated genes related to DNA replication (29.4%), DNA repair (18.8%), the cell cycle (14.5%), and cellular metabolic processes (6.7%). In contrast, MAC (B) displayed an upregulation of genes involved in defense against viruses (22.4%), bacteria (17.8%), and fungi (13%).

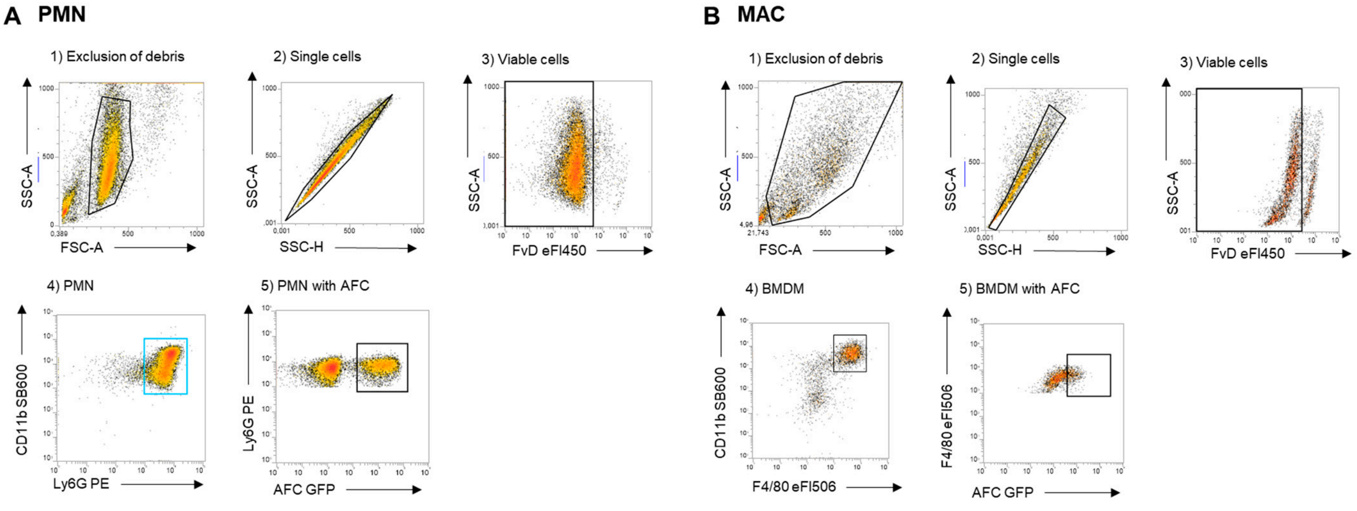


Figure S7. Gating strategy to evaluated phagocytic uptake of GFP-labeled AFC for PMN (A) and MAC (B).

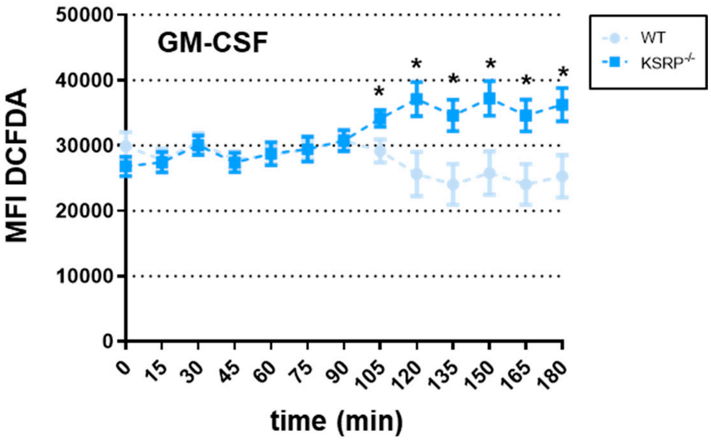


Figure S8. PMN from KSRP-/- mice produced significantly higher levels of ROS following stimulation.

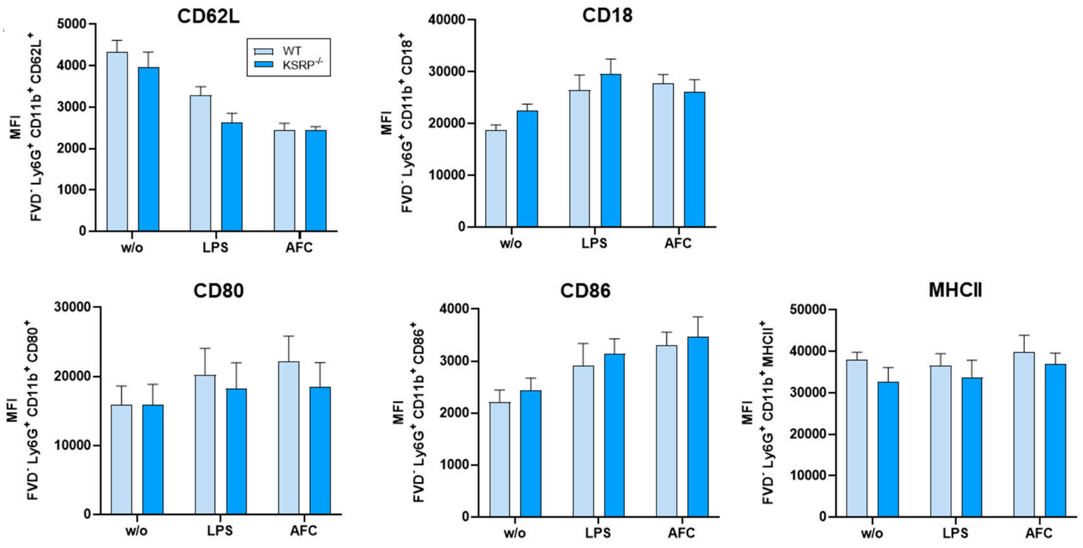


Figure S9. Stimulation of bone-marrow-derived PMN from WT and KSRP-/- mice has no genotype-dependent impact on (early) surface activation marker expression.

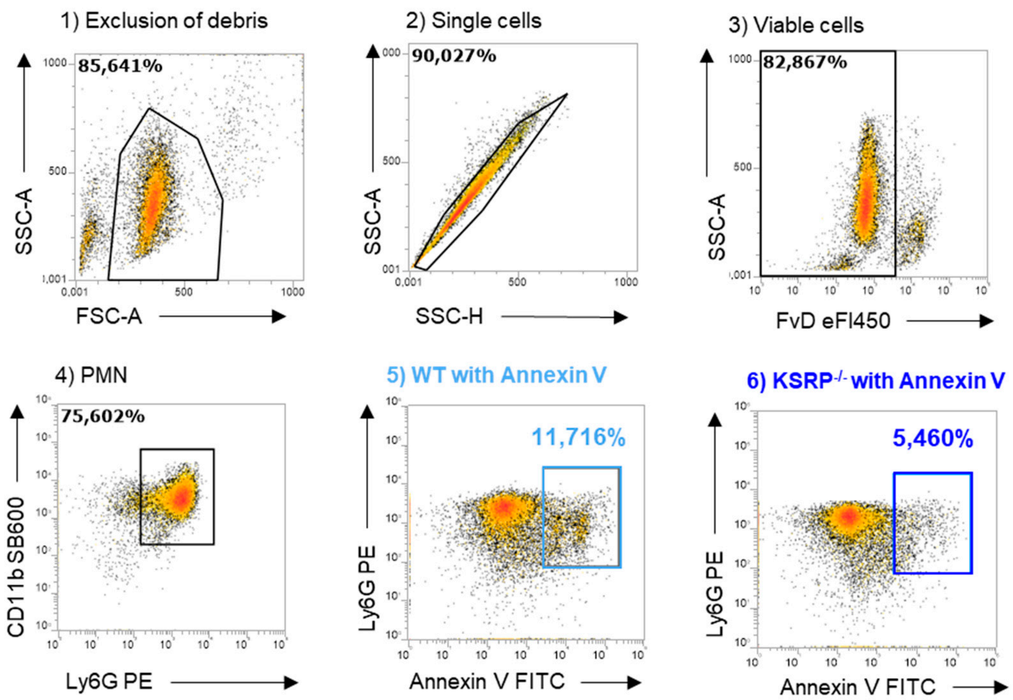


Figure S10. Gating strategy to evaluated apoptosis levels of PMN in WT and KSRP-/- mice.
